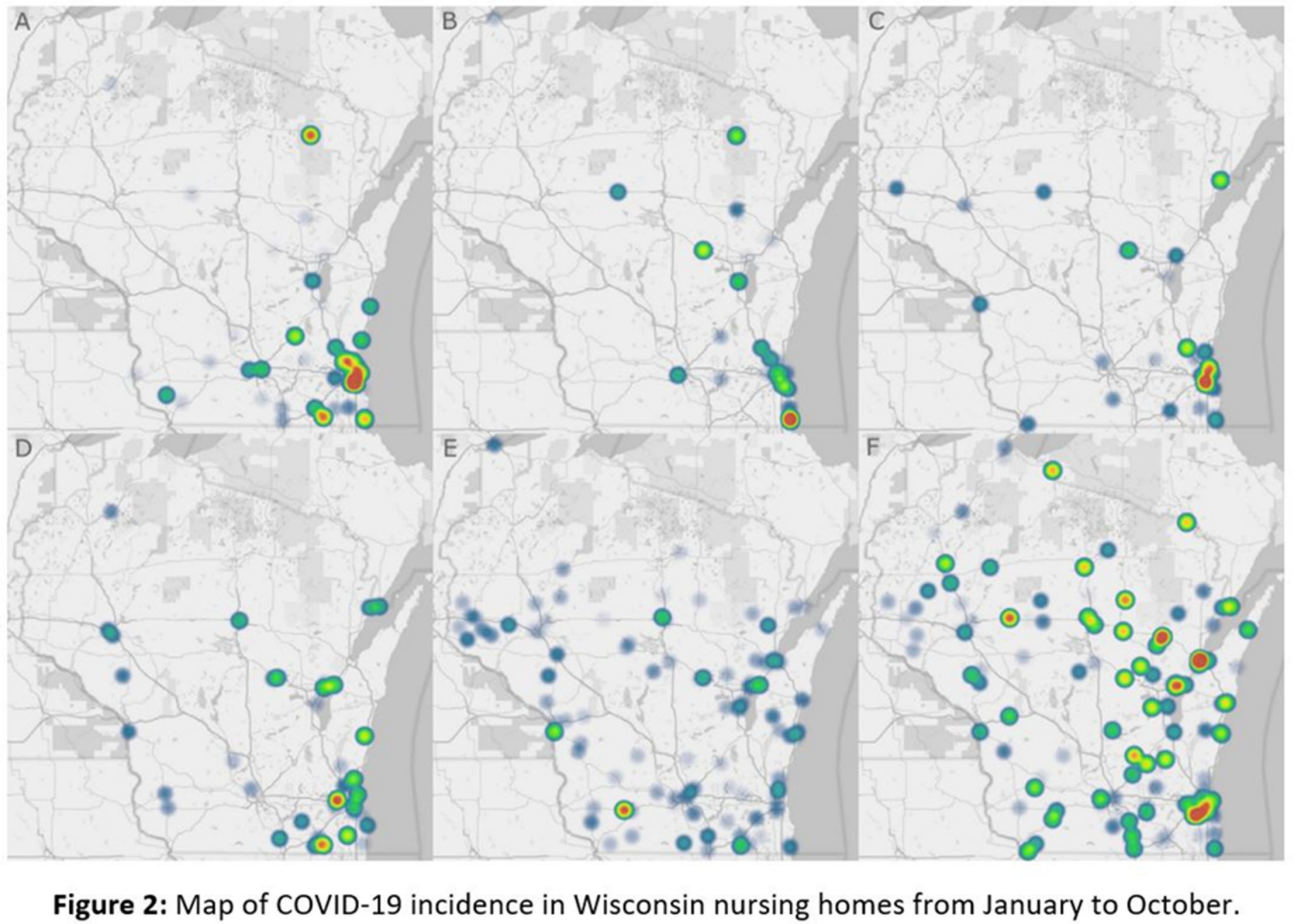# Coronavirus in Wisconsin Nursing Homes: A Longitudinal Analysis of the First 10 Months of the Pandemic

**DOI:** 10.1017/ash.2021.22

**Published:** 2021-07-29

**Authors:** Cameron Gmehlin, Frida Rivera, Jorge Ramos-Castaneda, Liliana Pezzin, EDmund Duthie, L. Silvia Munoz-Price

## Abstract

**Background:** The COVID-19 pandemic has disproportionately affected nursing home residents, and emerging evidence suggests quality, location, resident demographics, and staffing levels may be related to COVID-19 incidence within facilities. We describe the distribution of COVID-19 cases in Wisconsin nursing homes from January 2020 to October 2020, the effect of rural versus urban locations on COVID-19 incidence, and the temporal changes in COVID-19 incidence. **Methods:** We constructed a database using the Center for Medicaid and Medicare Services’ (CMS) publicly available data. Variables obtained per facility included location, number of beds, ownership type, average census, 5-star ratings (overall, quality, health, staffing, and nurse staffing categories), number of COVID-19 cases, resident Medicaid/Medicare share, area deprivation index, and social vulnerability index. Nursing homes were divided into tertiles based on total COVID-19 cases for descriptive analysis (zero cases, 1–7 cases, >7 cases). Demographic and clinical variables were reported as frequencies, mean (standard deviation) or median (interquartile range). We compared groups using the Pearson χ^2^ test and the Kruskal-Wallis test. COVID-19 incidence rates were calculated by dividing the number of COVID-19 cases by monthly occupied bed days and multiplied by 10,000. **Results:** From January 1, 2020, to November 1, 2020, in total, 3,133 SARS-CoV-2–confirmed cases were reported among 248 (70.5%) nursing homes. Urban location (*P* = .027), overall 5-star rating (P = .035), number of beds (p < 0.001), and average count of residents per day (p < 0.001) were associated with a greater number of COVID-19 cases. Temporal analysis showed that the highest incidence rates of COVID-19 in NHs were observed from January to May and in October 2020 (11.36 and 30.33 cases per 10,000 occupied-bed days, respectively). Urban NHs experienced higher incidence rates until September, then incidence rates among rural facilities surged (Fig.1A). In the first half of the year, NHs with lower quality scores (1-3 stars) had a higher COVID-19 incidence rate; however, in August this trend reversed, and facilities with higher quality scores (4-5 stars) showed the highest incidence rates (Fig.1B). Fig. 2 shows a temporal depiction of the shift from urban to rural settings. **Conclusions:** Higher COVID-19 incidence rates during the first 5 months of the pandemic were observed in urban, larger facilities with lower 5-star rating. By the end of the year, nursing homes in rural areas and those with higher quality ratings had the highest incidence rates.

**Funding:** No

**Disclosures:** None

**Figure 1.**
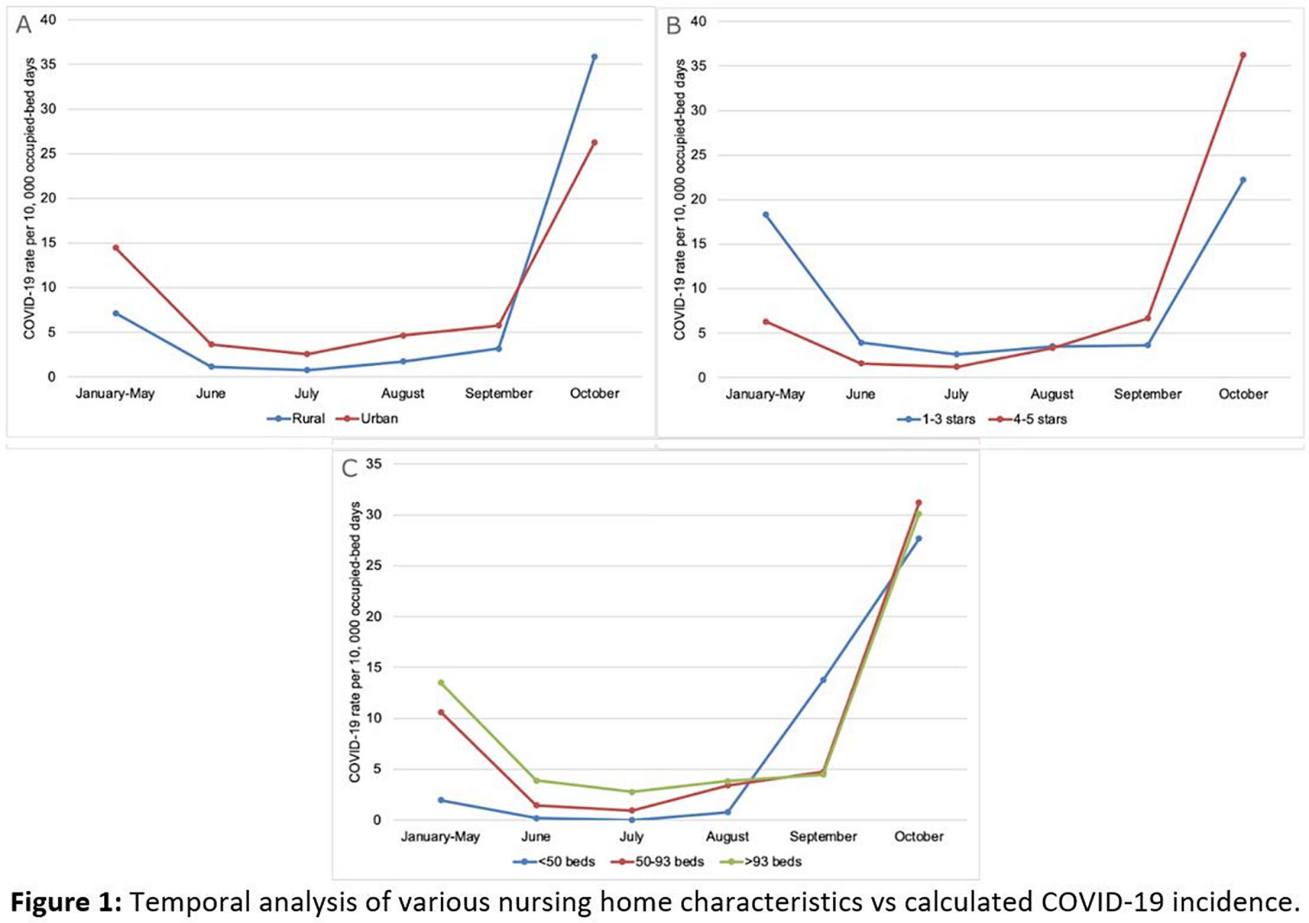

**Figure 2.**